# Death from Nitrous Oxide Gas and Ether

**Published:** 1877-06

**Authors:** 


### EDITORIAL, ETC.

*Deaths from Nitrous Oxide Gas and Ether.*—We have received from Dr. S. Parsons Shaw, of Manchester, England, a full description of the following case of death from Nitrous Oxide Gas, which appears in the June Number of this Journal. The following brief account is from the *Medical Times and Gazette*, of London :

"An inquest was held last week at Manchester on the body of Mr. George Morley Harrison, aged fifty-three, a surgeon in good practice, and formerly Lecturer on Medical Jurisprudence at the Manchester Royal School of Medicine, who died whilst under the influence of nitrous oxide gas, administered at his own request previous to having a tooth extracted by a neighbouring dentist. Mr. Harrison, it appears, being unnerved and excited, partly from the suffering he had undergone, and partly owing to the want of proper food, which the condition of his mouth had prevented him from taking, insisted on the inhalation being pushed until he should snore, and—for, at any rate, part of the time—held the mouthpiece in his own hand, and inspired very vigorously. The first attempt at extraction was made before he was fully insensible, and was abandoned until more of the gas had been given. Eventually, however, two teeth were removed. The patient did not appear to be coming round properly after the operation, and the dentist, taking alarm, sent for medical assistance. On the arrival of a surgeon, Mr. Harrison was pronounced to be quite dead. At the post-mortem examination there was found some fat about the heart; the cavities on the right side were distended with blood, while those on the left side were empty. The lungs on both sides were gorged with dark blood. All the other organs were healthy.

The jury came to the conclusion that the deceased "died from syncope during the administration of nitrous oxide gas for the extraction of teeth whilst labouring under fatty degeneration of the heart."

Very few fatal accidents have hitherto attended the use of nitrous oxide as an anæsthetic; hence it has come to be looked upon as almost free from danger. For minor operations it is administered every day, and often by persons who would shrink from the responsibility of giving chloroform. Dental practitioners especially use nitrous oxide very largely. A catastrophe such as has just taken place at Manchester serves to remind us that nitrous oxide can no more be said to be absolutely free from risk than any other anæsthetic with which we are acquainted. No opportunity, then, should be neglected of ascertaining the mode of action of nitrous oxide when it destroys life. In the only other fatal case which, so far as I know, has been recorded in England—I refer to the Exeter case—no inspection of the body was made. It becomes doubly important, therefore, that any fresh case should be reported with the utmost accuracy.

Now, judging from the facts of the case stated above, and especially from the condition of the thoracic viscera, found after death, it cannot, I think, be doubted that the jury were mistaken in their verdict, and that the case was one of death from asphyxia, not from syncope.

The question is of the highest scientific and practical importance. It is most undesirable that nitrous oxide should be credited with a paralysing influence over the heart, if it possesses no such influence. While again, looking at it from a practical point of view, we want to know in what direction we are to be on the look out for the first signs of approaching danger, and it will not do for our attention to be concentrated on the condition of the pulse and the circulation, if the real danger is from suffocation. It is stated that, in order to shield the dentist from blame, the medical witnesses somewhat encouraged the opinion which the jury embodied in their verdict. This however, I should be sorry to believe. I prefer to think that the jury, with the want of intelligence which is popularly ascribed to coroners' juries, misunderstood the drift of the medical evidence and returned their misleading verdict in spite of it."

The same Journal also records the death of a woman aged 55 years, from Ether and Nitrous Oxide Gas combined, which occurred at the Union College Hospital, the anæsthetics being administered for strangulated femoral hernia. Clover's apparatus was used. At the autopsy, stercoraceous matter was found in the trachea and right bronchus.

